# Fine-tuning mesoporous silica properties by a dual-template ratio as TiO_2_ support for dye photodegradation booster

**DOI:** 10.1016/j.heliyon.2023.e16275

**Published:** 2023-05-16

**Authors:** Maria Ulfa, Cindy Nur Anggreani, Novia Amalia Sholeha

**Affiliations:** a Chemistry Education Study Program, Faculty of Teacher Training and Education, Sebelas Maret University, Jl. Ir. Sutami 36A, Surakarta 57126, Indonesia; b College of Vocational Studies, Bogor Agricultural University (IPB University), Jalan Kumbang No. 14, Bogor 16151, Indonesia

**Keywords:** TiO_2_, CTAB, Silica mesoporous, Methylene blue, Photodegradation

## Abstract

Titanium dioxide (TiO_2_) has been integrated into the surface of mesoporous silica (SMG) synthesized via the hydrothermal approach and a dual template CTAB-Gelatin. XRD, nitrogen adsorption, FTIR, SEM-EDX, and UV–Vis DR spectroscopy were performed to evaluate a 1 wt% TiO_2_/SMG material. After titania incorporation, the addition of gelatin during the synthesis of SMG increases the pore volume to 0.76 cc/g. The expansion of the silica pores is caused by the development of TiO_2_ crystal grains on the mesoporous silica-gelatin. An increase in the gelatin-CTAB to mesoporous silica weight ratio modifies the surface area, pore size, and particle size without compromising the meso-structure. In this research, the TiO_2_/SMG composite demonstrated much greater photodegradability for methylene blue (MB) than the TiO_2_/mesoporous silica sample without gelatin. The experimental results indicate that the photocatalytic activity of methylene blue from SMG titania/silica samples is reliant on the adsorption ability of the composite and the photocatalytic activity of titania, with optimal activity from samples with the highest surface area and pore volume, which directly increase the Ti: Si ratio and decrease the photodegradability of the composite when the ratio is too high or too low.

## 1 Introduction

The presence of waste containing methylene blue is one of the most significant concerns in the world's oceans today [1]. Diverse efforts have been made to eliminate this waste because its adverse impacts on the health and growth of aquatic biota jeopardize the future availability of clean water [2]. Numerous attempts were undertaken, including adsorption techniques and photocatalysts. Photocatalyst is regarded as a promising clean approach for reducing methyl blue waste without accumulating residual compounds [2,3]. TiO_2_ is a commonly used metal in photocatalysts. However, when applied, pure TiO_2_ readily agglomerates, lowering the photodegradation efficiency by up to 80% [4]. Therefore, the good support material is required to ensure that TiO_2_ is properly distributed and effective for photodegradation [5]. Mesoporous silica is among the most preferred support materials [[6], [7], [8]].

Mesoporous silica has drawn the interest of researchers in recent years due to its multiple features, including inertness, controllability, large pore size, high surface area, high pore volume, and uniform morphology [[9], [10], [11], [12]]. All of these benefits of mesoporous silica are advantageous for numerous applications in adsorption, photocatalysis, separation, and energy storage [9,13,14]. Considering the high cost of hard templates and the complexity of the synthesis procedure, several researchers have selected the soft template technique, which utilizes synthetic surfactants as the major structure-directing agent in the synthesis of mesoporous silica [[15], [16], [17]]. The extensive use of synthetic surfactants such as P123, P127, CTAB, TMOH, and CTAC. Recently, CTAB was widely used in the synthesis process due to their self-assembly method into cylindrical-shaped, positively charged micellar then reacts with the negatively charged silica source, exchanging, releasing the counter anions of the surfactant and permits the modulation of silica structure [18]. Haynes et al. [19], proposed the Pluronic F127/P123 and CTAB as neutral and ionic soft templates to synthesize mesoporous silica. As a cosurfactant in the P123 system, CTAB directed the creation of large-pore mesoporous silica microspheres with tunable pore diameters. Moreover, the introduction of CTAB into the SBA-15 gel solution may result in the production of secondary micelles within the primary SBA-15 framework [19]. Another study, Nguyen et al. [20] also used CTAB as positively charged surfactant with various non-ionic surfactants i.e., Tween 20, Tween 80 and Brij S10 as co-template in the preparation of mesoporous silica nanoparticles (HMSN).

The utilization of synthetic surfactant: silica source ratio of approximately 1:2 (w/w) has an impact on the high costs required for large-scale silica synthesis, despite the fact that these surfactants or soft templates are removed through decomposition at the end of the stage [17,[21], [22], [23], [24]]. The usage of synthetic templates in large quantities is expensive, limiting the long-term production of large-scale mesoporous silica. Consequently, numerous attempts have been made to resolve this issue, including the natural template approach. Natural templates such as starch, gelatin, natural rubber, and gum Arabic are notable advances in the effort to limit the use of synthetic templates [[25], [26], [27], [28], [29], [30], [31], [32], [33]]. Gelatin has been discovered to be an effective structure-directing agent when combined with synthetic templates in a dual template strategy. Numpilai et al. [34] uses gelatin with ratio to silica source of 0–1.8 in the hydrothermal process to produce hollow porous silica spheres with a controlled shell thickness of 80–160 nm. The recent studies revealed that P123 and F127, when combined with gelatin, generated regular mesoporous silica and carbon with good application performance [12,35,36] with regular hexagonal pores of 104–320 Å and high surface area of 69–104 m^2^. However, no specific investigation has been conducted on the impacts of CTAB-gelatin dual template on mesoporous silica formation. Therefore, this study will employ a soft template of CTAB with gelatin to synthesize large pore and adjustable pore diameters of mesoporous silica subsequent TiO_2_ doping for methylene blue photodegradation.

## 2 Experimental

### 2.1 Material

The materials used in this research are HCl (37%, Sigma-Aldrich Merck KGaA, Mr 36.5 g/mol), H_2_O (pure analysis, Smart-Lab, Mr 18 g/mol), cetyl trimethylammonium bromide (CTAB, Sigma-Aldrich Merck KGaA, Mr 364.45 g/mol), Gelatin (Gelatin, Mr 90,000 g/mol), tetraethyl orthosilicate (TEOS, Sigma-Aldrich Merck KGaA, Mr 208.33 g/mol), titanium tetraisopropoxide (TTIP, Sigma-Aldrich Merck KGaA, Mr 204 g/mol), ethanol (Sigma-Aldrich Merck KGaA (Mr 46.068 g/mol), NaOH (reagent grade, ≥98%, pellets (anhydrous), Sigma-Aldrich Merck KGaA, Mr 39.997 g/mol), and methylene blue (C_16_H_18_C_l_N_3_S · xH_2_O, Sigma-Aldrich Merck KGaA, Mr 319.85 g/mol).

### 2.2 Synthesis mesoporous silica doped with titania

The limitation of this study is the mesoporous silica synthesis as support material from dual templated CTAB-gelatin in a specific ratio (Table 1) in 480 ml of 0.015 M NaOH at 80 °C with stirring. The resultant mixture was added to 5 ml of TEOS placed in an autoclave at 80 °C for hydrothermal reaction for 24 h. To remove residual surfactant, the product was filtered, washed with distilled water, dried at 70 °C for 24 h, and then calcined at 550 °C for 5 h at a rate of 2 °C/min. SMG_x_-H is the outcome obtained, where x is the gelatin to CTAB ratio and H is the hydrothermal procedure. 0.024 ml of titanium isopropoxide dissolved in 20 ml of *n*-hexane was added to 0.99 g of SMG_x_-H while stirring at 45 °C for 16 h, followed by drying at 160 °C for 2 h. The mixture was then dissolved in hexane, followed by 45 min of drying at 80 °C and 5 h of calcination at 550 °C to produce TiO_2_/SMG-x-H.

**Table 1:** Samples notation.

Samples	Gelatin (g)	CTAB (g)	TiO_2_ (g)
SMG_1_/TiO_2_ H	1	100	1
SMG_10_/TiO_2_ H	10	100	1
SMG_20_/TiO_2_ H	20	100	1
SMG_100_/TiO_2_ H	100	0	1
SMC_100_/TiO_2_ H	0	100	1

The instruments used to analyze the samples included the X-Ray Diffraction (Pananalytical XRD, PW3050/60, copper anode, and 0.02°/s scanning rate), Fourier Transform Infrared (Shimadzu FTIR spectrophotometer) with a resolution of 0.5 cm^−1^, Scanning Electron Microscopy-Energy Dispersive X- Ray (SEM-EDX, JEOL JSM-700 microscope) at a voltage speed of 15.0 kV, and Brunauer-Emmett-Teller (BET, Quantachrome Nova 1200e. The crystal size of mesoporous silica nanoparticles with the Debye Scherrer formula is as in Eq. (1) below:

Eq. 1 $$D=\frac{0.9\;\lambda }{B\;\cos\;\theta }$$

D is the crystal size in Ǻ, λ is the wavelength used in the XRD test, which is 1.54056 Å, and B is the width of half the peak in radians. θ is the angular position of the peak formation. XRD results also show FWHM to be able to determine the value of B (rad) and cyrstallinity (Eq. (2)).

Eq. 2 $$Crystallinity\;\left(\%\right)=\frac{crystalline\;peak\;area}{crystalline\;and\;amorphous\;peak\;areas}\times 100\%$$

### 2.3 Photodegradation of methylene blue

TiO_2_/SMG_x_-H photocatalyst with mass of 50 mg was added to 10 ml of 5 ppm methylene blue, that had followed by a closed shaker procedure (dark adsorption) for 30 min in triplicate and then UV irradiation. Using a UV–Vis spectrophotometer with a wavelength range of 670 nm, absorbance readings were taken every 10 min. Analysis of the percent degradation of methylene blue using a UV–Vis spectrophotometer by observing the absorbance curve and determining its value using the %ED formula (Eq. 3). The kinetic study also is carried out by using first order (Eq. (4)) and second order (Eq. (5)) reaction.

Eq. 3 $$Degradation\;efficiency\;of\;MB\;\left(\%\;ED\right)=\frac{C_{o}-C_{t}}{C_{o}}\times 100\%$$

Eq. 4 $$C_{0}-C_{t}=kt$$

Eq. 5 $$\ln\frac{C_{0}}{C_{t}}=-kt$$

Where $C_{o}=$ initial concentration of MB dan $C_{t}=$ concentration of MB at t minutes.

## 3 Result and discussion

**Fig. 1:**
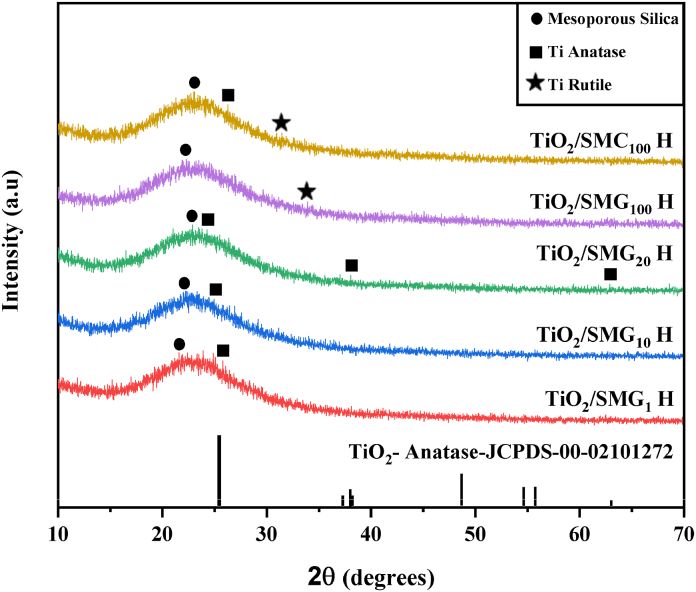
XRD results of the samples.

The XRD profiles of each sample at 2θ = 10–70° are shown in Fig. 1. The diffraction peaks centered at 2θ of 25.3° correspond to (002) planes of SiO_2_ and the hump is confirming the amorphous nature of SiO_2_. The peak is in accordance with the previous literature that amorphous silica has a diffraction peak of 2θ = 21–25° (JCPDS No. 29–0085) [37]Utilization of gelatin or CTAB as a single template results in less crystalline silica than using dual template gelatin-CTAB. Dual template CTAB-gelatin in silica formation increases crystallinity by approximately 7% and does not significantly alter the crystallite size, as indicated in Table 2. However, due to the limited proportion of Ti in silica, no sample exhibited a significant peak of anatase phase on TiO_2_ (JCPDS No. 2101272) [5,38,39]. The presence TiO_2_ on the mesoporous silica was confirmed by the FTIR and EDS data.

**Table 2:** Elemental analysis by EDS.

Sample	% w			
	C	O	Si	Ti
SMG_1_/TiO_2_ H	9.52	46.05	43.92	0.51

SMG_10_/TiO_2_ H	8.14	51.07	40.47	0.32

SMG_20_/TiO_2_ H	7.01	52.79	39.85	0.34

SMG_100_/TiO_2_ H	19.52	53.71	26.52	0.25

SMC_100_/TiO_2_ H	–	52.03	47.72	0.25

**Fig. 2:**
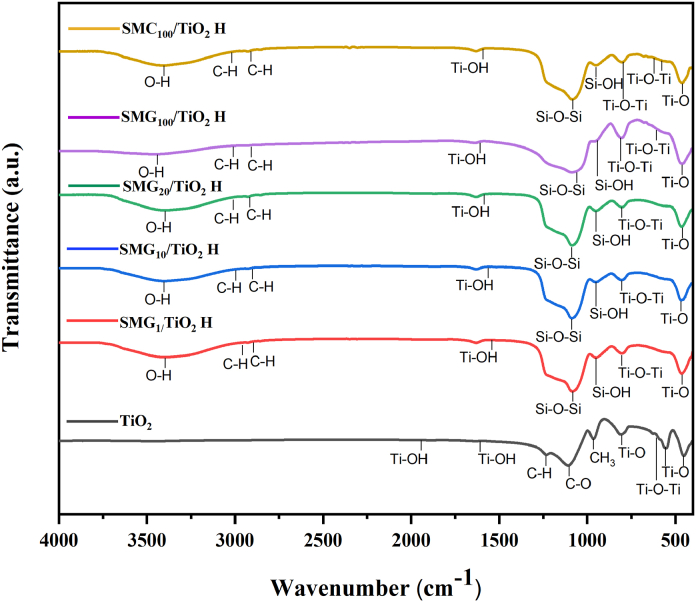
FTIR analysis of samples.

The FTIR of SMG_x_-H/TiO_2_ with different gelatin concentrations was depicted in Fig. 2. All samples exhibited wide absorption band at wavenumber of 3369–3454 cm^−1^ as the hydroxyl (-OH). The peak at 1050, 800, and 949-963 cm^−1^ revealed the *Si*–*O*–Si symmetric, asymmetric bending vibrations, and Si–OH symmetric vibration, respectively [3]. The samples with high gelatin concentrations have slightly higher intensity of hydroxyl and *Si*–*O*–Si absorption peak due to the high involvement of gelatin in the structure formation process. Moreover, the titania presence in the silica mesoporous was evidence by the peak at wavenumber of 1627–1633 cm^−1^ as Ti–OH bending [40] and at 803-806 cm^−1^ as Ti–*O*–Ti bond [41]. The wavenumber peak at this research are consistent with those of earlier research [3,6,7,21,[42], [43], [44]].

**Fig. 3:**
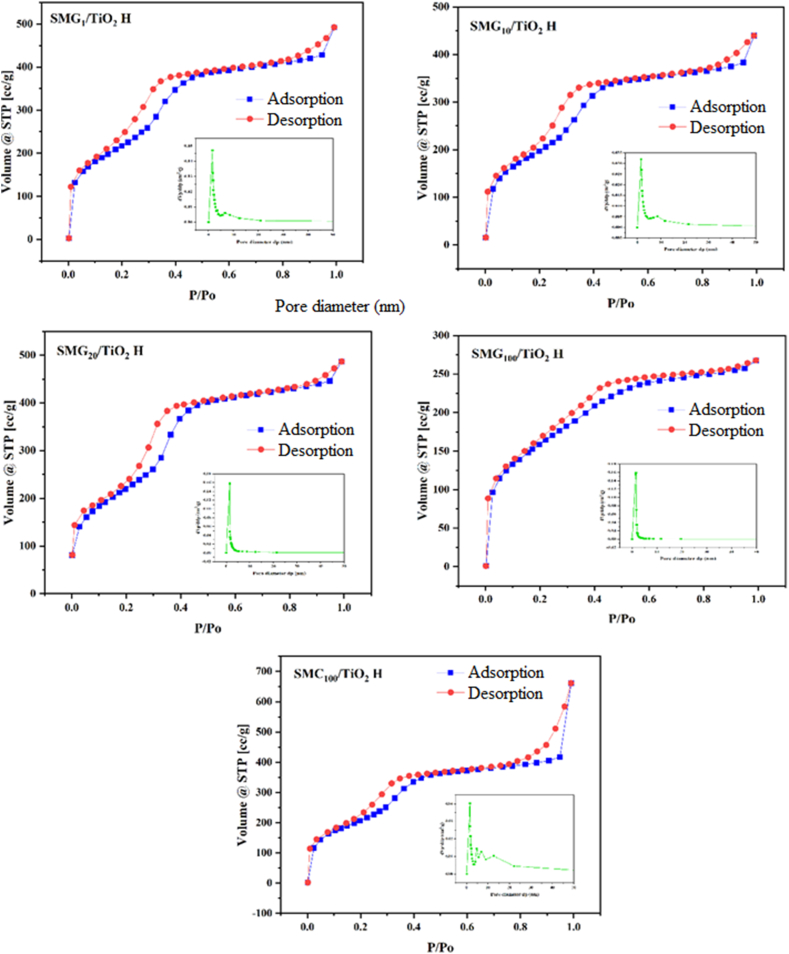
N_2_ adsorption-desorption of (a) SMG_1_/TiO_2_ H, (b) SMG_20_/TiO_2_ H, (c) SMC_100_/TiO_2_ H, (d) SMG_10_/TiO_2_ H, and (e) SMG_100_/TiO_2_ H.

Not only FTIR (Fig. 2), the presence TiO_2_ on the mesoporous silica was confirmed by EDS data (Table 2). The EDX spectra of all sample demonstrated the presence of TiO_2_ and SiO_2_ as the minority and majority component, respectively. The presence of TiO_2_ is stable at low concentration from 0.25 to 0.51% w/w which show the succesfully the incorporation process. Increasing gelatin content in the samples led to the decreasing Si content due to the replacing Si by C which may be ascribed to the particle decomposition of carbon. By comparing the carbon contents, we found that not only a higher C content corresponded to larger aggregates on the surface but also led to the decreasing surface area which confirmed by nitrogen adsorption desorption analysis (Fig. 3 and Table 3).

**Table 3:** Physicochemical properties of samples.

Samples	S_BET_ (m^2^/g)	S_micro_^a^ (m^2^/g)	S_meso_^b^ (m^2^/g)	V_micro_^a^ (cc/g)	V_meso_^b^ (cc/g)	V_total_ (cc/g)	D^b^ (nm)	%Ti	DP^c^ (μm)	CS^d^ (nm)	C^e^ (%)	ED^f^ (%)
TiO_2_/SMG-_1_H	769.09	0.02	769.00	0.29	0.39	0.68	3.72	0.51	0.11	0.49	69.31	89.58
TiO_2_/SMG-_10_H	799.89	0.01	799.90	0.26	0.50	0.76	3.58	0.32	0.09	0.48	73.71	95.81
TiO_2_/SMG-_20_-H	791.93	0.03	791.90	0.35	0.40	0.75	3.70	0.25	0.11	0.49	70.60	91.28
TiO_2_/SMG-_100_-H	577.46	0.04	577.50	0.07	0.34	0.41	5.36	0.34	0.12	0.49	67.80	83.23
TiO_2_/SMC_100_-H	763.23	0.03	763.20	0.32	0.36	0.68	3.84	0.25	0.11	0.49	68.01	88.22

a S_micro_ and V_micro_ determined by t-plot method.

b S_meso_, V_meso_, and pore diameter (D) determined by BJH method.

c DP = diameter of particle by SEM.

d CS = crystallites size determined by XRD.

e C = crystallinity of samples determined by XRD.

f ED = the photodegradation efficiency of MB.

The analysis of N_2_ adsorption-desorption (Fig. 3 a-e) depicts that all samples have isotherm type-IV with H-1 hysteresis, in which the hysteresis has a narrow loop. The type-IV isotherm exhibited the adsorption and desorption branches are almost vertical and almost parallel with the inflection position which lies at the relative pressure (P/P_o_) in the range of 0.4–0.9 as indicative of mesoporous samples. Based on Table 3 as the summarized of the textural properties, the surface area of TiO_2_-impregnated silica produced from 100% gelatin (SMG_100_-H/TiO_2_) and 100% CTAB (SMC_100_-H/TiO_2_) was 577 m^2^/g and 763 m^2^/g, respectively. When dual template gelatin-CTAB technique with 1–20% w/w gelatin was used, the surface area increased as much as 5% compared to SMC_100_-H/TiO_2_ and 22% compared to SMG_100_-H/TiO_2_ to 799.89% for SMG_10_-H/TiO_2_. The pore volume of TiO_2_ impregnated on mesoporous silica as supported synthesized from CTAB-gelatin increased by roughly 12%, 0.67 cc/g to 0.76 cc/g for SMG_10_-H/TiO_2_. The increment of surface area and pore volume was the synergetic effect of CTAB-gelatin which occurs when the polar groups of both molecules engaged strongly with Si in the precursor thus controlled the pore formation.

**Fig. 4:**
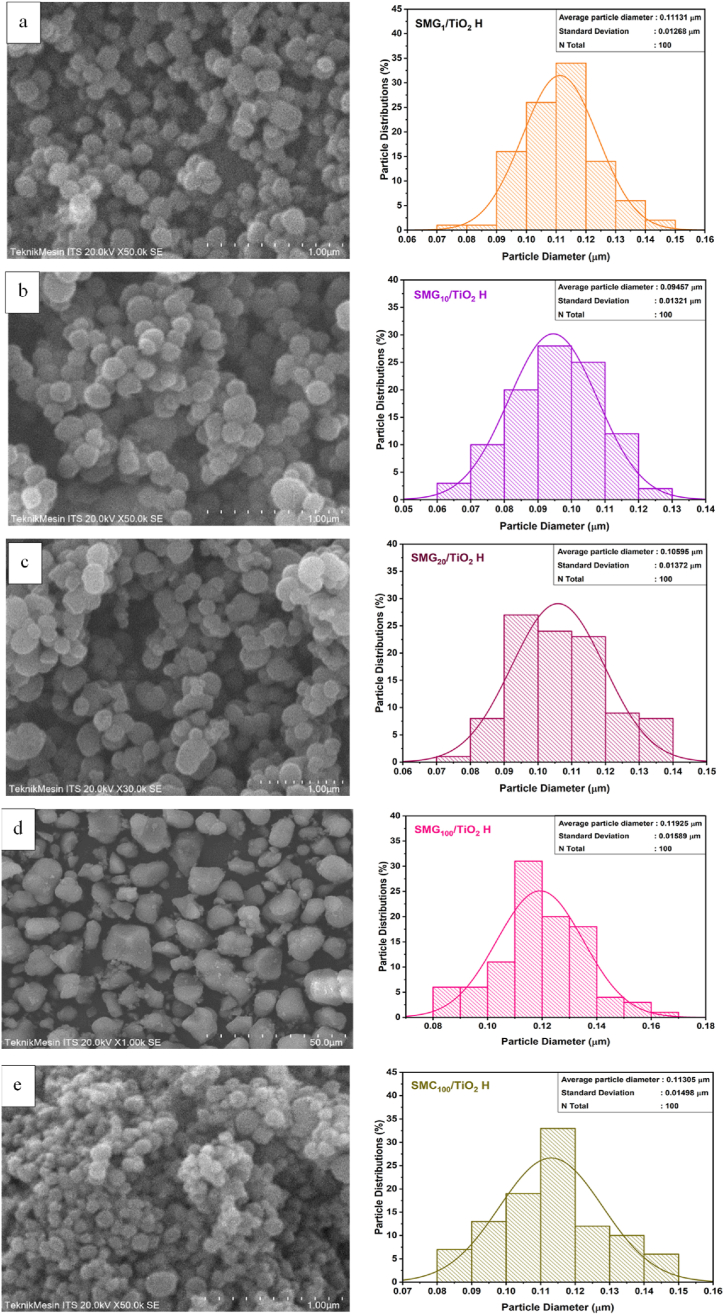
SEM analysis of (a) SMG_1_/TiO_2_ H, (b) SMG_10_/TiO_2_ H, (c) SMG_20_/TiO_2_ H, (d) SMG_100_/TiO_2_ H, and (e) SMC_100_/TiO_2_ H.

Fig. 4(a–e) displayed the SEM analysis and particle size distribution of mesoporous silica at varying concentrations of gelatin-CTAB as support of TiO_2_. The particle size of silica reduced with increasing gelatin concentration up to 10%, from 0.11 to 0.095 μm, before increasing to 0.12 with the addition of 20% gelatin. The inclusion of gelatin of 1–20% alters the morphology of silica, which, in the absence of gelatin, tends to form dense granule aggregates in the shape of big aggregates. The negatively charged silica causes aggregation of positively charged amine gelatin to produce a gel network that controls the particle formation.

**Fig. 5:**
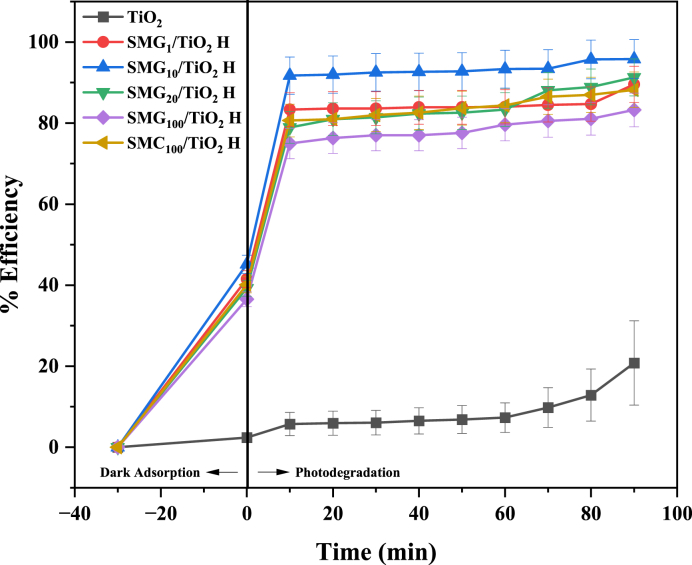
Photocatalysis results of TiO_2_/mesoporous silica samples with various concentrations of gelatin.

The mesoporous silica as support for TiO_2_ was used as photocatalyst to degrade methylene blue. According to Fig. 5, the degradation of methylene blue accelerates significantly with increasing UV exposure time at 5–40 min of irradiation and remains steady at 40–90 min. Exposure time affects the methylene blue degradation as the color of the solution fades, the UV light is easier to reach the photocatalyst. When irradiation time extends, the photon energy absorbed by the TiO_2_/mesoporous silica photocatalyst on the surface rises, more methylene blue will be degraded. In addition to photocatalytic, the dark adsorption process was conducted in the dark for 30 min prior to the irradiation process to homogenize the solution and reach equilibrium. Therefore, the methylene blue solution degrades more quickly when irradiated with UV since some of the methylene blue molecules have been trapped on the surface of the mesoporous silica as booster material. Due to adsorption in the dark, the concentration of methylene blue decreased by 0 min of irradiation time. The findings is consent with investigations that employed silica as a supporting material for titania in the photodegradation of methylene blue [[45], [46], [47], [48]].

**Fig. 6:**
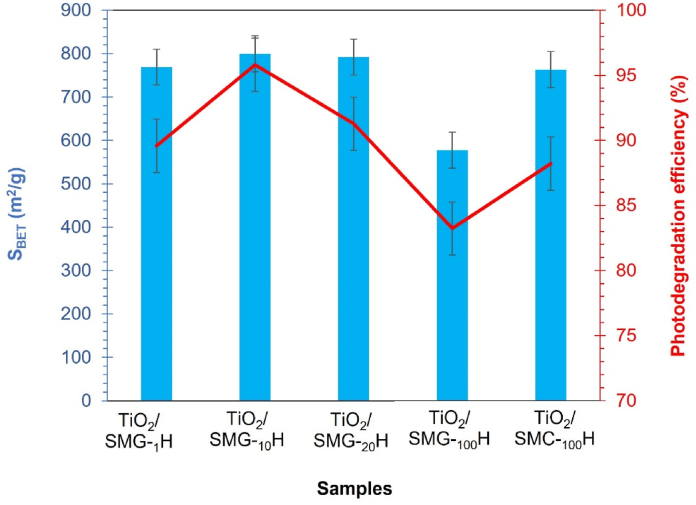
Correlation textural properties in TiO_2_/mesoporous silica for methylene blue photodegradation efficiency

The SMG_10_H/TiO_2_ would degrade the methylene blue at the highest rate, 95.81%, whereas TiO_2_ degraded the least, at a rate of 94.80%. Increasing the concentration of gelatin to 10% improves the degradation of methylene blue, which occurs due to the participation of gelatin-CTAB to generate a structure with a large surface area and an even particle size distribution. The high degradation rate also influenced by highest surface area of 799.89 m^2^/g with a particle size of 0.095 μm of the SMG_10_H/TiO_2_. The degradation efficiency of TiO_2_/mesoporous silica photocatalysts by single template (SMG_100_H/TiO_2_ and SMC_100_H/TiO_2_) is significantly lower than that of TiO_2_/mesoporous silica photocatalysts from dual template. Without the synergetic effect CTAB-gelatin, TiO_2_ lacks mesoporous silica as support material to aid in the dispersion of particles, causing agglomeration that limits photodegradation. The correlation between surface area and the degradation efficiency of material was displayed in Fig. 6. The degradation efficiency is also compared to other studies (Table 4). Among graphene oxide and other silica material, the SMG_10_H/TiO_2_ demonstrated the high efficiency due to their high surface area and pore volume.

**Table 4:** Photodegradation of methylene blue by silica based catalysts.

Samples	Catalyst weight (g)	S_BET_ (m^2^/g)	V_total__pore_ (cm^3^/g)	Time (min)	MB amount	Photodegradation efficiency (%)	References
Ni complex/mesoporous silica	–	–	–	15	10 ppm	92.00	[49]
GO/hollow mesoporous silica	0.02	879.91	0.73	10	20 ppm	93.00	[50]
CaTiO_3_/g-C_3_N_4_	0.01	–	–	120	10 ppm	92.70	[51]
TiO_2_ NPs	0.03	–	–	120	20 ppm	65.00	[52]
TiO_2_@rGO	0.04	121.48	2.95	40	0.01 mM	51.30	[53]
TiO_2_/SiO_2_ (S1T)	–	467.00	0.56	60	0.5 mM	79.00	[54]
20TiO_2_/SiO_2_	0.20	55.45	0.52	40	20 ppm	99.00	[55]
TiO_2_/SiO_2_-800	0.30	141.60	0.23	30	10 ppm	80.00	[56]
ZnPc-SiO_2_-TiO_2_	0.10	–	–	60	50 ml	80.00	[57]
*N*-doped TiO_2_/CTAB/SiO_2_	0.10	157.70	–	300	100 ml 10^−5^ M	30.00	[58]
TiO_2_/SiO_2_-500	0.30	89.80	–	120	20 ppm	60.00	[59]
TiO_2_/SMG_10_ H	0.05	799.89	0.76	10	5 ppm	95.81	**This study**

**Fig. 7:**
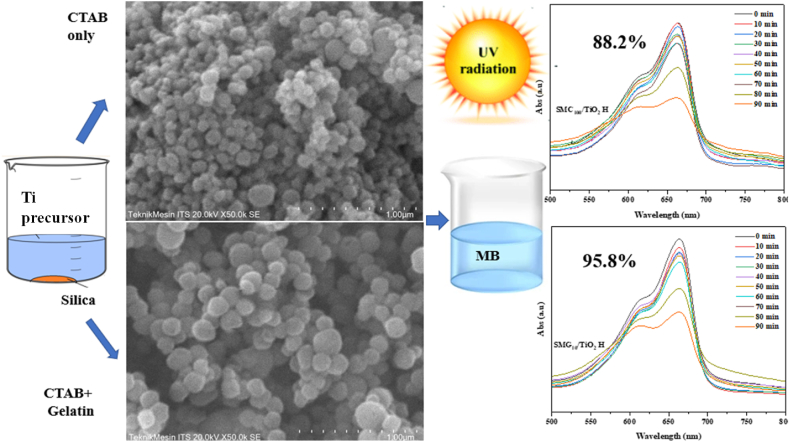
Illustration of titania impregnation on Mesoporous Silica using gelatin template.

The schematic illustration of CTAB-gelatin role for mesoporous silica formation as support in TiO_2_ displayed in Fig. 7. CTAB has a polar group CTA^+^ while gelatin has an NH^+^ group; both have a high affinity for Si because to the electronegativity difference, which exceeds 1.2. When the polar groups of gelatin and CTAB fight to interact with Si, the non-polar groups of gelatin and CTAB repel Si. Due to the carbon chains from the non-polar CTAB and gelatin groups, this causes the space framework of the silica particles to lengthen. Because gelatin with a molecular weight of 400 KDa [60] has a substantially longer chain than CTAB with a molecular weight of approximately 0.4 KDa [[61], [62], [63]], the TiO_2_-impregnated silica generated by including gelatin-CTAB has a greater pore capacity than that synthesized from CTAB or gelatin alone. Gelatin can induce the condensation of silica precursors via hydrogen bonding or electrostatic interactions between –NH_3_^+^ and silanol species, as well as CTA^+^ from CTAB interacting with silanol from Si precursors. Gelatin also contributes to the expansion of the silica core and the aggregation of silica particles, a chain aggregation process that is crucial to the generation of the morphology of mesoporous materials. The alteration of pore volume and surface area affect the methylene blue photodegradation.

The reaction mechanism for the degradation of methylene blue on TiO_2_ and TiO_2_/mesoporous silica photocatalysts when exposed to UV light is as follows (Eqs. Eq. 4, Eq. 5, Eq. 6, Eq. 7, Eq. 8) [64]:

Eq. 4 TiO_2_ + hv → TiO_2_ (e^−^ + h^+^)

Eq. 5 OH^−^ + h^+^ → •OH

Eq. 6 H_2_O + h^+^ → •OH + H^+^

Eq. 7 OH + Methylene blue → CO_2_ + H_2_O

Eq. 8 Methylene blue + e^−^ → CO_2_ + H_2_O

When TiO_2_ and TiO_2_/mesoporous silica photocatalysts as booster material absorb photons with energy larger than or equal to the TiO_2_ band gap energy width, the electrons in TiO_2_ are stimulated from the valence band to the conduction band. On the surface of the photocatalyst, electrons (e^−^) as conduction band and holes (h^+^) as valence band vacancies are created as the material absorbs light energy. The e^−^ and h^+^ can stimulate the redox reaction of O_2_ and H_2_O to produce superoxide radical (•O_2_) at −0.33 eV and hydroxyl radical (•OH) at 1.99 eV [51]. These two substances can migrate to the surface of TiO_2_ and TiO_2_/mesoporous silica, where they will undergo redox reactions with organic contaminants, in this case methylene blue. Hole electrons will react with OH^−^ to form hydroxyl radicals (•OH). This radical is an extremely powerful oxidizing agent and the primary oxidizer in the photocatalytic oxidation of methylene blue to carbon dioxide, water, and other mineralization byproducts. Meanwhile, electrons (e^−^) will react with methylene blue to form CO_2_ and H_2_O as reduction products [35,[65], [66], [67]].

**Fig. 8:**
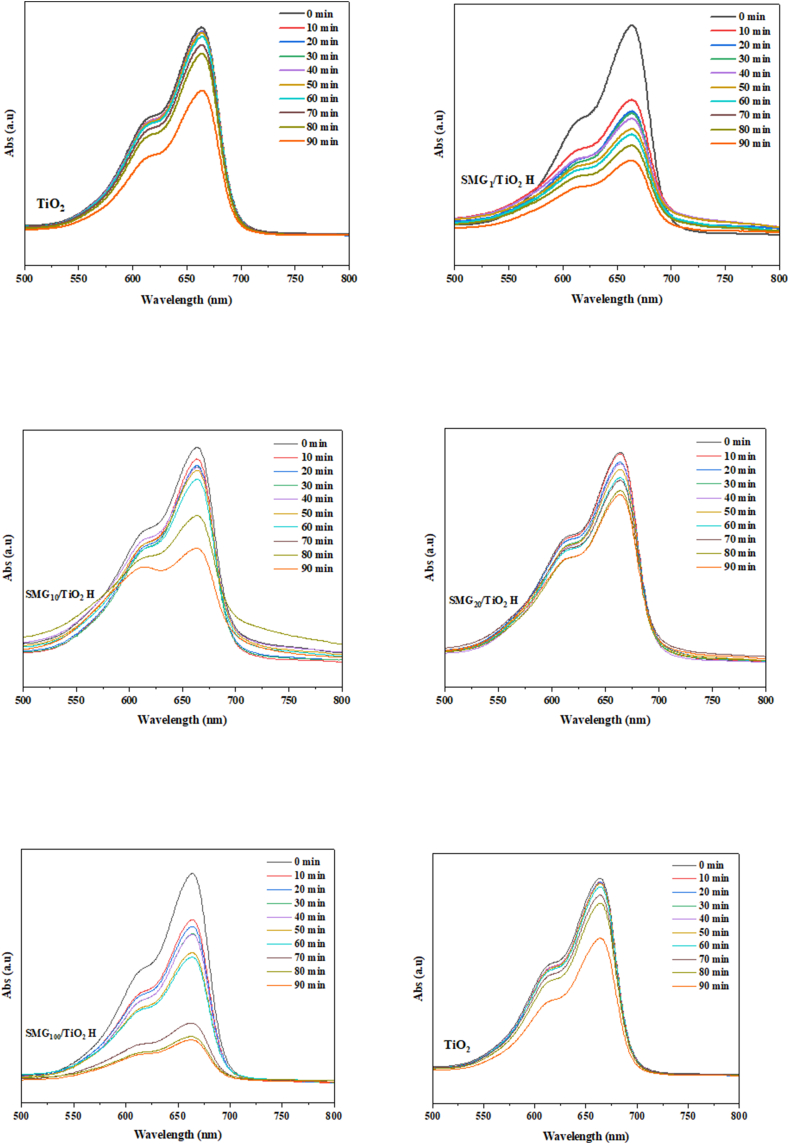
UV–Vis spectra of methylene blue solution degraded by TiO_2_ and SMG/TiO_2_ based photocatalyst a) TiO_2_, b) SMG_10_/TiO_2_ H, c) SMG_100_/TiO_2_ H, d) SMG_1_/TiO_2_ H, e) SMG_20_/TiO_2_ H, and f) SMC_100_/TiO_2_ H

**Fig. 9:**
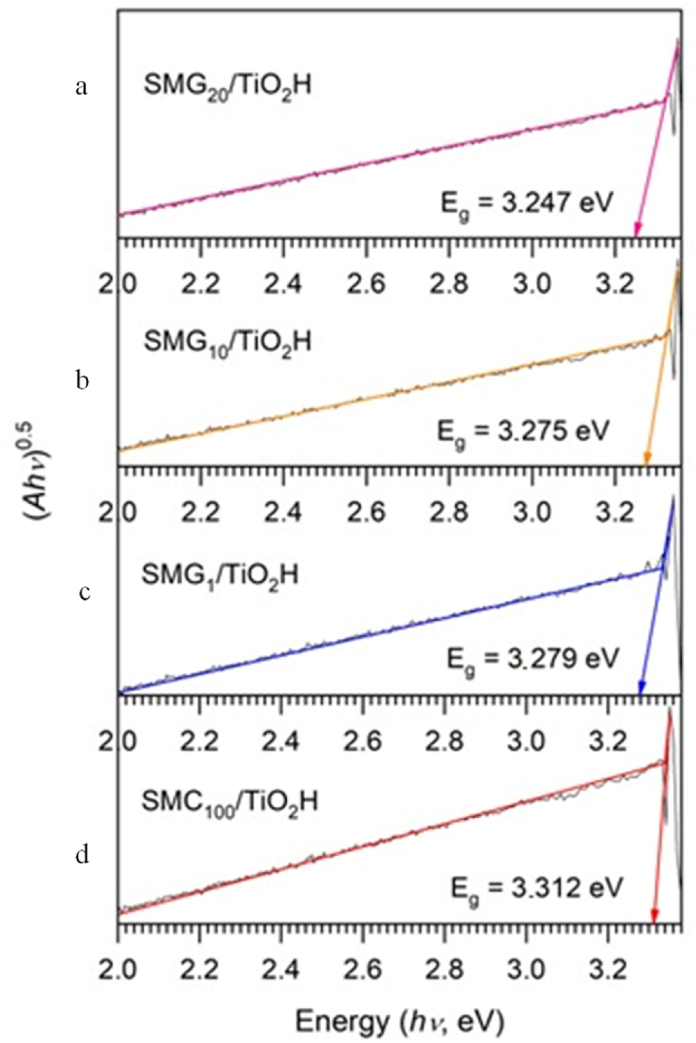
The band gap energy of all sample silica after titania impregnation a) SMG_20_/TiO_2_ H, b) SMG_10_/TiO_2_ H, c) SMG_1_/TiO_2_ H, and d) SMC_100_/TiO_2_ H.

TiO_2_ content also influenced the band gap energy of each silica sample. The band gap energy increases as the particle size of semiconductor materials decreases, as predicted by the model theory of the cohesive energy of the confinement effect [68]. The band gap of the samples was estimated by analyzing diffuse reflectance spectra (DRS) in the UV–vis range (Fig. 8). The plots of the band gap energy of silica following titania impregnation and the calculated band gap energy were shown in Fig. 9. The material band energy gap fell as its gelatin content increased, reaching a maximum of 3.23 eV for samples containing 20% gelatin. The values did not vary linearly with gelatin concentration, therefore a smaller crystallite size at 10% gelatin (4.8 nm) represents a decrease in band gap energy. The non-uniform dispersion of TiO_2_ particles on the silica surface may account for this inconsistency.

**Fig. 10:**
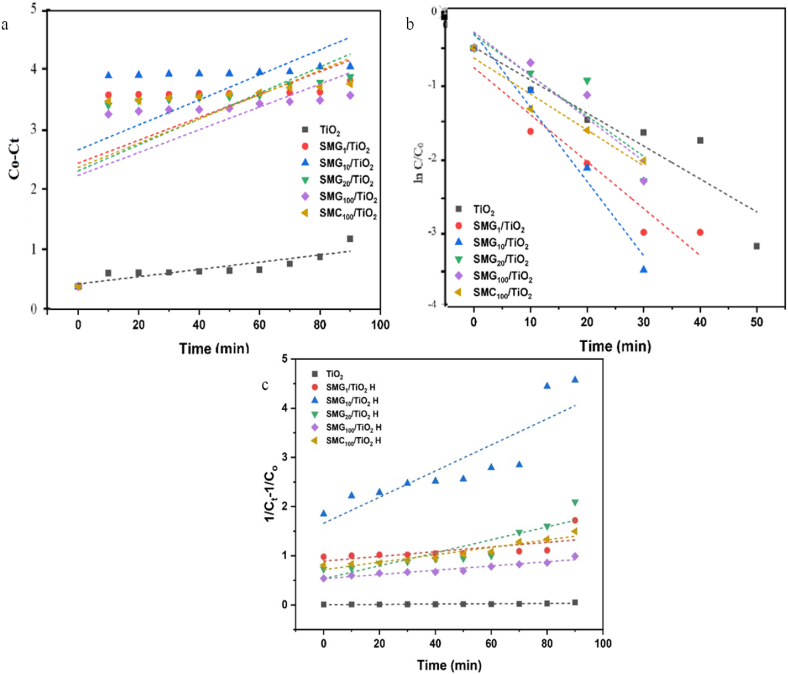
Kinetics of methylene blue photodegradation by a) pseudo-zero order, b) Pseudo First order model, and c) pseudo second order

Fig. 10 depicts the pseudo zero, first order, and second order kinetics models of methylene blue decomposition. During this investigation, these models were applied to each of the catalyst samples that were analyzed. The coefficients of determination (R^2^) that were obtained based on the various models utilized to fit the kinetic data are presented in Table 5. Due to the assumption that the rate-limiting step is the chemical sorption of MB as target molecules and that the oxidation occurs via photo-induced electron transfer between the reactants and photoactive particles, the photodegradation of the entire sample is more accurately described by the pseudo first order model with R close to 0.99. Aforementioned assumption that the rate-limiting step is chemical sorption of MB as target molecules [55,69,70].

**Table 5:** Kinetic of Methylene Blue Photodegradation all sample.

Sample	C_o_ (ppm)	ED (%)	Pseudo Zero Order			Pseudo First Order			Pseudo Second Order		
			Kinetic constant (10^−4^)	Initial rate (mg/L/min)	R^2^	Kinetic constant (10^−3^)	Initial rate (mg/L/min)	R^2^	Kinetic constant (10^−3^)	Initial rate (mg/L/min)	R^2^
TiO_2_	5	20.79	6.50	0.14	0.63	14.03	0.31	0.79	0.34	0.04	0.58
SMG_1_/TiO_2_ H	5	89.58	2.20	4.12	0.47	1.65	0.55	0.44	4.78	0.89	0.37
SMG_10_/TiO_2_ H	5	95.81	2.60	4.53	0.88	2.99	0.85	0.84	26.56	1.66	0.74
SMG_20_/TiO_2_ H	5	91.27	6.80	3.87	0.91	4.86	0.38	0.88	13.20	0.53	0.77
SMG_100_/TiO_2_ H	5	83.23	4.90	3.68	0.96	3.01	0.28	0.95	4.32	0.53	0.92
SMC_100_/TiO_2_ H	5	88.22	4.60	3.97	0.97	3.18	0.45	0.96	7.55	0.72	0.91

The measured values of k as the kinetic constant degradation process by pseudo first order model are the best fit for all samples. SMG_10_/TiO_2_–H has the highest degradation rate constant from the decomposition of methylene blue among all catalysts based on kinetic constant and initial rate value. Rate constant is almost 2.7 times higher than pure TiO_2_^.^ The increase in photocatalytic activity for SMG_10_/TiO_2_–H (Table 4) is due to the enhanced surface plasmonic resonance effect of dispersed TiO_2_ on the surface of mesoporous silica under visible light as well as an electron storage space for degradation of methylene blue which allows the separation of charge carriers. These result which was in good agreement with the previous photogenerated carrier migration rate of the catalysts research [51,[71], [72], [73]]. Comparative photocatalytic degradation revealed the superiority of TiO_2_ compared to catalysts reported elsewhere.

## 4 Conclusion

Combining hydrothermal and physical techniques, dual template gelatin-CTAB was used to produce TiO_2_/mesoporous silica as an accelerator material for methylene blue photodegradation using TiO_2_/mesoporous silica. Prior to the inclusion of 20% gelatin, the particle size of silica decreased from 0.11 to 0.095 m with increasing gelatin concentrations up to 10%. In addition to particle size, the gelatin-CTAB ratio had a negligible impact on the sample's surface area, pore volume, and pore diameter. The mesostructured anatase TiO_2_ was relatively dispersed in accordance with the 799.89 m^2^/g silica surface area. Titania on mesoporous silica without gelatin addition (SMC) exhibited significantly lower MB photodegradability (88.2%) than titania on mesoporous silica with gelatin addition (SMG) (95.81%).

## Author contribution statement

Maria Ulfa: Conceived and designed the experiments; Contributed reagents, materials, analysis tools or data; Wrote the paper.

Cindy Nur Anggreani: Performed the experiments; Wrote the paper.

Novia Amalia Sholeha: Analyzed and interpreted the data; Wrote the paper.

## Data availability statement

Data will be made available on request.

## Declaration of competing interest

The authors declare that they have no known competing financial interests or personal relationships that could have appeared to influence the work reported in this paper
